# A long-range interactive DNA methylation marker panel for the promoters of HOXA9 and HOXA10 predicts survival in breast cancer patients

**DOI:** 10.1186/s13148-017-0373-z

**Published:** 2017-07-24

**Authors:** Seong-Min Park, Eun-Young Choi, Mingyun Bae, Jung Kyoon Choi, Youn-Jae Kim

**Affiliations:** 1 Translational Research Branch, Research Institute, National Cancer Center, Goyang, Gyeonggi 10408 Republic of Korea; 2 Personalized Genomic Medicine Research Center, KRIBB, Daejeon, 34141 Republic of Korea; 3 Department of Bio and Brain Engineering, KAIST, Daejeon, 34141 Republic of Korea

**Keywords:** Biomarker, Prognosis, DNA methylation, Survival, Long-range interaction, Chromatin interaction, HOXA9, HOXA10

## Abstract

**Background:**

Most DNA cancer methylation markers are based on the transcriptional regulation of the promoter-gene relationship. Recently, the importance of long-range interactions between distal CpGs and target genes has been revealed. Here, we attempted to identify methylation markers for breast cancer that interact with distant genes.

**Results:**

We performed integrated analysis using chromatin interactome data, methylome data, transcriptome data, and clinical information for breast cancer from public databases. Using the chromatin interactome and methylome data, we defined CpG-distant target gene relationships. After determining the differences in methylation between tumor and paired normal samples, the survival association, and the correlation between CpG methylation and distant target gene expression, we selected CpG methylation marker candidates. Using Cox proportional hazards models, we combined the selected markers and evaluated the prognostic model. We identified six methylation markers in HOXA9 and HOXA10 promoter regions and their long-range target genes. We experimentally validated the chromatin interactions, methylation status, and transcriptional regulation. A prognostic model showed that the combination of six methylation markers was highly associated with poor survival in independent datasets. According to our multivariate analysis, the prognostic model showed significantly better prognostic ability than other histological and molecular markers.

**Conclusions:**

The combination of long-range interacting HOXA9 and HOXA10 promoter CpGs predicted the survival of breast cancer patients, providing a comprehensive and novel approach for discovering new methylation markers.

**Electronic supplementary material:**

The online version of this article (doi:10.1186/s13148-017-0373-z) contains supplementary material, which is available to authorized users.

## Background

Breast cancer is both the most common cancer and the most frequent cause of cancer-related deaths among women [1]. Based on the expression level of hormone receptors, such as the estrogen receptor (ER) and progesterone receptor (PR), or human epidermal growth factor receptor (Her2), breast cancers are divided into several subtypes, and small molecules or antibodies targeting ER, PR, and Her2 have been used in breast cancer therapies [2]. Breast cancer is conventionally diagnosed by mammography, but this method cannot be applied to some cases, including women with premenopausal breast cancer [3]. Molecular markers and reference laboratory tests for breast cancer diagnosis and prognosis have been developed, but the methods are limited to specific subtypes, such as node-negative and ER-positive breast cancer [4, 5]. Thus, novel approaches for the diagnosis and prognosis of breast cancer are still needed.

DNA methylation is one of the most well-known aberrations in human cancers [6]. During tumor progression from normal tissue to invasive cancer, the total level of DNA methylation gradually decreases, but the frequency of hypermethylated CpG islands on promoters increases, causing the transcriptional silencing of tumor-suppressive genes [7, 8]. DNA methylation markers have advantages compared to other molecular markers. For example, hypermethylation of promoter CpGs is a common and early event during the progression of various tumors [9, 10], and DNA methylation is more chemically and biologically stable than RNA or most proteins [6]. DNA methylation markers for cancer diagnosis and prognosis have been discovered, and some of them have been used in clinical trials [11, 12]. For breast cancer, researchers have also reported particular DNA methylation markers [13, 14], some of which need further development for clinical application.

The DNA methylation of promoters and CpG islands is known to inhibit target gene expression by regulating the binding of transcription modulators to the promoter [15, 16]. The long-range interaction between CpGs and target genes has been reported [17, 18]. A recent genomic study revealed that the correlation between DNA methylation at distal regulatory sites and long-range target gene expression is significantly stronger than the correlation with promoter methylation and that differences in DNA methylation between cancer and normal tissues at distal regulatory sites are significantly greater than differences in promoter methylation among various cancer types [19]. Nevertheless, most DNA methylation markers for cancer diagnosis and prognosis have been developed based on promoter-gene relationships because of the difficulty of defining the relationship between distal CpGs and target genes. The long-range action of distal CpG-target gene interaction and transcriptional regulation can be specified by chromatin interactome data, particularly data from RNA polymerase II (Pol II) chromatin interaction analysis by paired-end tag sequencing (ChIA-PET) [20]. Thus, novel approaches could be based on the long-range interaction between CpGs and their target genes.

In this study, we identified DNA methylation markers for breast cancer and the putative target genes that had long-range interactions using an integrated analysis incorporating the chromatin interactome, methylome, and transcriptome data for breast cancer from public databases. We tried to validate the chromatin interaction, methylation status, and transcriptional regulation. Selected marker candidates were combined to establish a prognostic model and evaluated as markers for breast cancer.

## Methods

### Public data analysis

The Cancer Genome Atlas (TCGA) methylome (Illumina Infinium Human Methylation 450k BeadChip microarray data, Infinium HM450k) and transcriptome (high-throughput RNA sequencing, RNA-seq) data containing clinical information were downloaded from the International Cancer Genome Consortium (ICGC) data portal (http://icgc.org/). The chromatin interactome (chromatin interaction analysis by paired-end tag sequencing, ChIA-PET) data were downloaded from the Encyclopedia of DNA Elements (ENCODE) databases (https://genome.ucsc.edu/ENCODE/). Another Infinium HM450k methylome dataset for validation was downloaded from the NCBI GEO database (http://www.ncbi.nlm.nih.gov/geo/) (GSE39004). The expression microarray (Affymetrix Human Genome U133 Plus 2.0 microarray, affyU133P2) data for MCF7 breast cancer cells after 5-azacytidine (5-aza C) treatment and the untreated control data were downloaded from the NCBI GEO database (GSE22250). The methylome data were globally normalized using *β* values (methylation ratio). The RNA-seq data were normalized based on the RPKM (reads per kilobase per million mapped reads) values. The affyU133P2 data were globally normalized using the Robust Multi-array Average (RMA) method.

The genomic positions were defined by the human hg19 reference genome. Genomic loci from 2000 bp upstream to 500 bp downstream of the transcription start sites (TSS) were defined as promoters. ENCODE MCF7 Pol II ChIA-PET data deposited in the UCSC genome browser database (http://genome.ucsc.edu/) were used to define CpG-target gene relationships. Genes whose promoters were anchored by ChIA PET reads were defined as target genes, and CpGs that overlapped with opposite ends of promoter-anchored ends were defined as distal CpGs.

Statistical tests were performed using the R program (https://www.r-project.org/). Graphs and heatmaps were prepared using Excel (Microsoft) and R.

### Cell culture and AZA treatment of the MCF7 breast cell line

The MCF7 cell line was purchased from the American Type Culture Collection (ATCC). MCF7 was maintained in complete Dulbecco’s modified Eagle medium (DMEM, HyClone) at 37 °C in a humidified 5% CO_2_ incubator. The complete medium was supplemented with 10% fetal bovine serum (HyClone), 100 U/ml penicillin/streptomycin (WelGENE), and 2 mM L-glutamine (HyClone).

The cells were treated with 1 μM 5-aza-2′-deoxycytidine (5-AZA C) (Sigma-Aldrich, A3656) dissolved in DMSO (Sigma-Aldrich, D2650), and the equivalent amount of DMSO was used as a control treatment. The cells were harvested after 72 h.

### Chromosome conformation capture (3C)

For this process, 5.0 × 10^6^ cells were cross-linked with 2% formaldehyde for 10 min at 25 °C. Five milliliters of NP-40 buffer (10 mM Tris-HCl, pH 7.5, 10 mM NaCl, 0.2% NP-40, and a protease inhibitor cocktail) was added to the cells, and the cells were incubated at 4 °C for 2 h. The mixture was then centrifuged, and the pellet was resuspended in 0.5 ml of 1.2× DpnII restriction enzyme buffer (NEB). Fifteen microliters of 10% SDS was added to the sample, and the mixture was incubated at 37 °C for 1 h. Forty microliters of 25% Triton-X100 was added, and the sample was incubated at 37 °C for 1 h. The sample was digested with 400 units of DpnII restriction enzyme at 37 °C for approximately 18 h. To deactivate DpnII, 80 μl of 10% SDS was added to the sample, and the mixture was incubated at 65 °C for 20 min. Then, 6.125 ml of 1.15× ligation buffer and 300 μl of 25% Triton-X100 were added, and the sample was incubated at 37 °C for 1 h. The digested samples were ligated with 100 units of T4 DNA ligase (Promega) at 16 °C for 4 h and then at 25 °C for 30 min. Reverse cross-linking and proteinase K treatment were performed overnight at 65 °C. The chromatin was then treated with RNase A for 1 h at 37 °C. The DNA was purified using a phenol/chloroform extraction or with a QIAquick PCR Purification Kit.

3C–PCR assays were performed using amfiXpand PCR Master Mix. The data were normalized to “internal” primers for the GAPDH gene. At least three independent biological replicates were included for each 3C–PCR assay. The primer sequences are listed in Additional file 1: Table S1.

### Reverse transcription PCR

The total RNA was extracted using the RNeasy Mini Kit (QIAGEN) according to the manufacturer’s instructions. Reverse transcription was performed with 1 μg of total RNA as the template and M-MLV Reverse Transcriptase (Promega). RT-PCR assays were performed using AmfiXpand PCR Master Mix (GenDEPOT). The cDNA expression was normalized to the levels of GAPDH. The primers used for the PCR reactions were designed either manually or using the Primer3 program (http://biotools.umassmed.edu/bioapps/primer3_www.cgi). All primer sequences are listed in Additional file 1: Table S1.

### Pyrosequencing

The total DNA was extracted using a QIAamp DNA Blood Mini Kit (QIAGEN) according to the manufacturer’s protocol. In total, 0.5 μg of total DNA from each of the samples was used for bisulfite conversion using an EZ DNA Methylation Lightning kit (Zymo Research). The bisulfite-converted DNA was amplified using TOPsimple Premix (Enzynomics). Pyrosequencing was performed using the PyroMark Q96 ID (PSQ 96MA, QIAGEN) system according to the manufacturer’s protocol. Pyrosequencing primers (forward, reverse, and sequencing) were designed using the PSQ Assay Design program (version 1.0.6). All primer sequences are listed in Additional file 1: Table S1.

### Code accessibility

We provided our Python and R scripts in GitHub (https://github.com/lastmhc/long-range_interactive_DNA_methylation_marker).

## Results

### HOXA9 and HOXA10 promoter CpG selection from public data

**Fig. 1:**
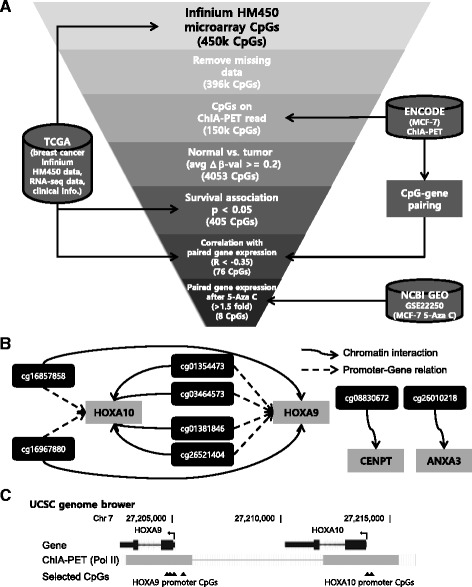
Selection of long-range interacting CpG methylation markers for the diagnosis and prognosis of breast cancer patients. **a** Scheme for the marker selection using pubic data (from the TCGA breast cancer dataset: Infinium 450k array data (methylome), RNA-seq data (transcriptome), and clinical information; from ENCODE: ChIA-PET data (chromatin interactome) for MCF7 breast cancer cells; from NCBI GEO: expression microarray data (transcriptome, GSE22250) for MCF7 breast cancer cells after 5-Aza C treatment). **b** Selected CpG marker candidates and long-range interacting genes. **c** Gene structure, the selected CpG loci and chromatin status near the HOXA9 and HOXA10 locus (UCSC genome browser)

To identify DNA methylation markers for breast cancer that physically interact with distant genes, we performed an integrated analysis and stepwise selection of the DNA methylation markers using publicly available chromatin interactome, methylome, transcriptome, and clinical information (Fig. 1a). The following data sets were used: the chromatin interaction analysis by paired-end tag sequencing (ChIA-PET) data for the MCF7 breast cancer cell line from the ENCODE database, Illumina Infinium Human Methylation 450k BeadChip (Infinium HM450k) microarray data, high-throughput RNA sequencing (RNA-seq) data and clinical information for breast cancer patients from TCGA database, and Affymetrix Human Genome U133 Plus 2.0 microarray (affyU133P2) data for MCF7 breast cancer cells after 5-aza C treatment from the NCBI GEO dataset (GSE22250). For the CpG sites probed by Infinium HM450k, CpGs that had missing values in the TCGA breast cancer dataset were removed. To select CpGs with long-range interactions, we used the MCF7 ChIA-PET data for RNA polymerase II (Pol II) and selected CpGs with genomic positions that overlapped with the ChIA-PET reads. Subsequent analyses were performed using the data from TCGA breast cancer patients who had both tumor and the paired solid normal tissue samples because we tried to select CpG methylation marker candidates that have both diagnostic and prognostic value. To determine the diagnostic value, the methylation difference of each CpG between the tumor and paired normal samples (Δ*β* value) was calculated, and hypermethylated CpGs (average Δ*β* > 0.2) were selected. To determine the prognostic value, after calculating the association between CpG methylation and the survival of breast cancer patients, we selected significantly associated CpGs (*p* < 0.05, log-rank test). To identify putative target genes of the selected CpGs, we paired the hypermethylated CpGs and distant gene promoters using the MCF7 Pol II ChIA-PET data. Using this information, we calculated the Pearson correlation coefficient (*R*) between the methylation of the CpGs and the expression of paired genes. To identify the putative relationship between the hypermethylated CpGs and distant target genes, we selected CpG-target gene relationships that had a highly negative correlation (*R* < −0.35). To test the DNA methylation sensitivity of the putative target genes, we examined the expression change in the genes after 5-aza C treatment using the GSE22250 dataset. Differentially expressed (fold change >1.5) genes (DEGs) and the paired CpGs were selected. Thus, eight putative CpG-target gene relationships that were supposed to compose long-range regulation modules were selected (Fig. 1b). Interestingly, six existed on Homeobox gene 9 and 10 (HOXA9 and 10) promoter loci, and four CpGs on the HOXA9 promoter and two CpGs on the HOXA10 promoter physically interacted with one another in the single ChIA-PET read (Fig. 1c). Thus, we subsequently focused on the methylation status of four CpGs on the HOXA9 promoter and two CpGs on the HOXA10 promoter.

### Long-range interplay of HOXA9 and HOXA10 promoter methylation markers

**Fig. 2:**
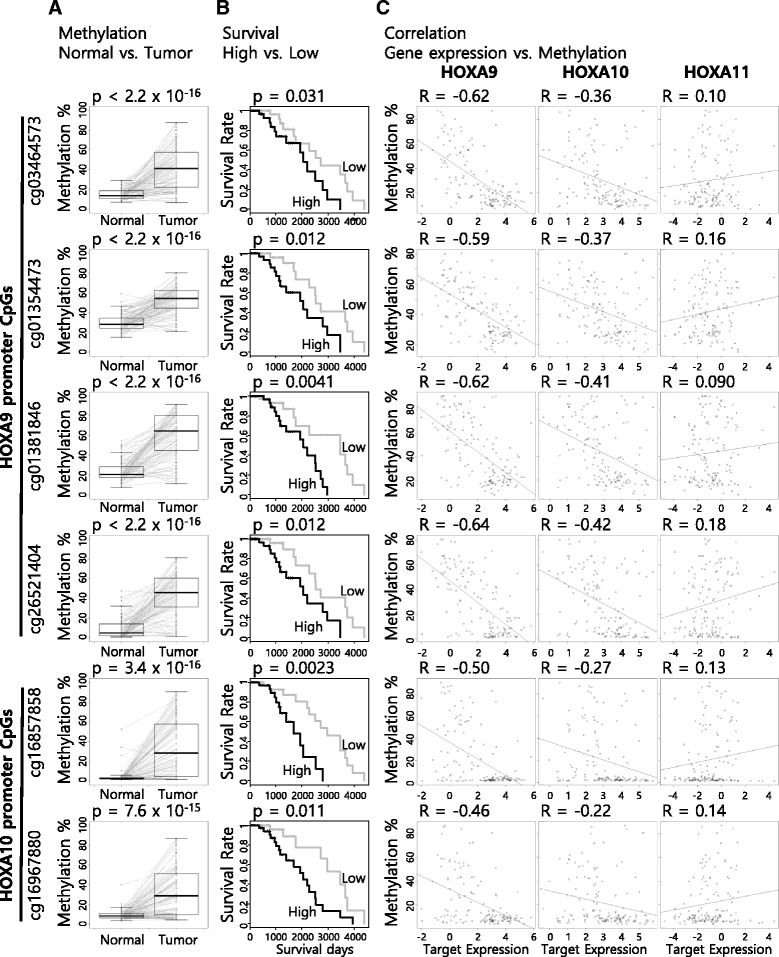
Estimation of CpGs on HOXA9 and HOXA10 promoters as diagnostic and prognostic markers for breast cancer. **a** Differences in the methylation status of the CpGs between tumor and paired normal tissues (diagnostic value). **b** Association between survival and the CpG methylation status (prognostic value, *black*: high, *gray*: low). **c** Correlation between methylation level of the CpGs and expression level of the HOXA9, HOXA10, and HOXA11 genes

HOXA9 and HOXA10 have been reported to be tumor suppressor genes in breast cancer [21–23]. The methylation of the HOXA9 and HOXA10 promoters is associated with cancer progression in various cancers [24, 25]. Previous analyses identified HOXA9 and HOXA10 promoter methylation marker candidates and their chromatin interactions. Thus, we further investigated the methylation status and the association with the survival of breast cancer patients using TCGA Infinium HM450k data and clinical information for paired tissues. First, we examined the DNA methylation differences between the normal and tumor samples. Comparing the DNA methylation percentage of each of the six CpGs on the HOXA9 and HOXA10 promoters in tumor tissues and their paired normal samples revealed that the methylation was significantly higher in the tumor samples than their paired normal samples (*p* < 1.0 × 10^−14^ for all six CpGs, *n* = 90, paired *t* test) (Fig. 2a). Using the survival data, we examined the association between the survival rate of breast cancer patients after surgery and the DNA methylation of each of the six CpGs on the HOXA9 and HOXA10 promoters. We divided the patients into two groups based on the median Δ*β* value. Survival was significantly lower in the patient group with higher methylation percentages than the patient group with lower methylation percentages (*p* < 0.05 for all six CpGs, *n* = 82, log-rank test). These results suggested that the six CpGs on the HOXA9 and HOXA10 promoters show possibility as diagnostic and prognostic markers for breast cancer.

After paring and integrating the TCGA Infinium HM450k and RNA-seq data, we investigated the target gene expression status, survival association, and correlation with neighbor genes. Comparing the expression level of HOXA9, HOXA10, and HOXA11 in tumors and their paired normal samples revealed that the expression of each HOXA9 and HOXA10 gene was significantly lower in the tumor samples than their paired normal samples (*p* = 1.3 × 10^−14^ for HOXA9, *p* = 7.6 × 10^−9^ for HOXA9, *n* = 49, paired *t* test) (Additional file 1: Figure S1A). Survival was not significantly associated with the expression of HOXA9, HOXA10, and HOXA11 (Additional file 1: Figure S1B). To define the CpG-target gene relationships, we examined the correlation among the six CpG methylations and the gene expression of HOXA9, HOXA10, and HOXA11 (Pearson correlation, *n* = 142). The methylation of all six CpGs was negatively correlated with the expression of HOXA9 (Fig. 2c). Interestingly, the methylation of the two HOXA10 promoter CpGs showed a higher correlation with HOXA9 expression than HOXA10 expression, whereas the four HOXA9 promoter CpGs highly correlated with HOXA9 expression (Fig. 2c). These results implied that the major target gene of the six CpGs is HOXA9 and that the two HOXA10 promoter CpGs regulate HOXA9 expression through a long-range interaction.

### Validation of the long-range interplay between the promoters of HOXA9 and HOXA10

**Fig. 3:**
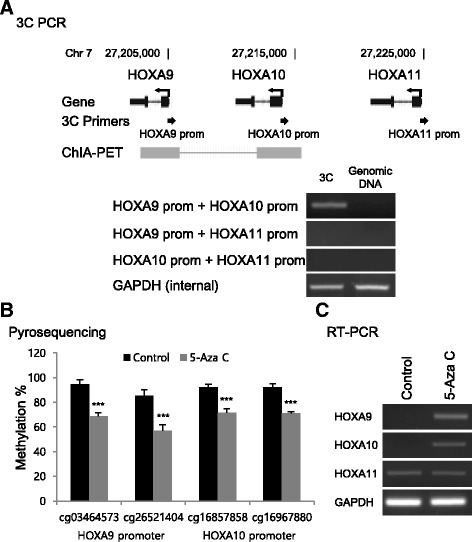
Validation of the CpG-gene relationship of HOXA9 and HOXA10 promoters. **a** Validation of chromatin interaction between HOXA9 and HOXA10 promoters using 3C PCR assays. **b** Validation of the HOXA9 and HOXA10 promoter CpG methylation decrease by 5-Aza C treatment. **c** Validation of the HOXA9 and HOXA10 gene expression increase by 5-Aza C treatment

Considering previous analyses, we hypothesized that CpGs on the HOXA9 and HOXA10 promoters interplayed through a long-range interaction. Using a cell-based model, we tried to validate the chromatin interaction between the HOXA9 and HOXA10 promoters. By performing 3C PCR assays in MCF7 and MDA-MB-231 breast cancer cells and MCF10A normal breast cells, we confirmed that the HOXA9 and HOXA10 promoters physically interacted each other, while the HOXA11 promoter did not interact with the HOXA9 or HOXA10 promoter (Fig. 3a and Additional file 1: Figure S2). Next, we tried to investigate whether the gene expression of HOXA9, HOXA10, and HOXA11 could be influenced by methylation status. We treated MCF7 breast cancer cells with 5-aza C during culture. Using a pyrosequencing assay with 5-aza C-treated cells and the controls, we confirmed that 5-aza C treatment decreased the methylation levels of four CpGs (Fig. 3b). Because we could not design good primers for two CpG sites on the HOXA9 promoter, the methylation of only two HOXA9 promoter CpG sites were validated. Consequently, we examined the gene expression change of HOXA9, HOXA10, and HOXA11 by performing a RT-PCR assay with 5-aza C-treated cells and the controls. We found that the expression of HOXA9 and HOXA10 increased with 5-aza C treatment, while the expression of HOXA11 did not (Fig. 3c). These results suggested that the CpGs on the HOXA9 and HOXA10 promoters regulated the target gene expression levels through long-range interactions.

### Prognostic value of HOXA9 and HOXA10 methylation marker combinations

**Fig. 4:**
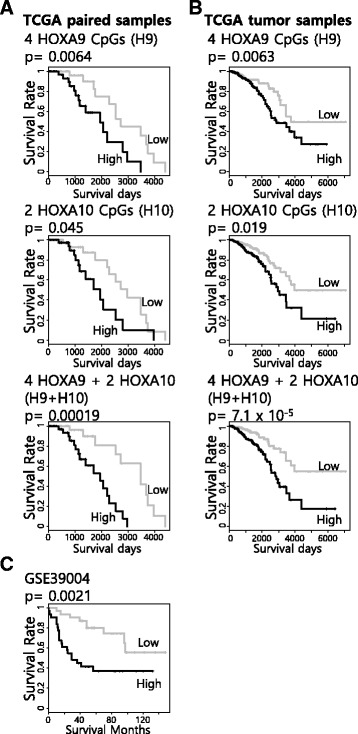
Combination of HOXA9 and HOXA10 promoter CpG methylation markers to enhance the prognostic ability. **a** Evaluation of the CpG combinations using the TCGA paired sample dataset. **b** Evaluation of the CpG combinations using the TCGA all sample dataset. **c** Evaluation of the combination of six HOXA9 and HOXA10 promoter CpGs using an independent dataset (NCBI GEO GSE39004) (*black*: high, *gray*: low)

As previously shown, the methylation of each HOXA9 and HOXA10 promoter CpG showed potential as a prognostic marker (Fig. 2b). To increase the prognostic ability, we tried to combine the CpGs and evaluated the prognostic abilities of the combinations. By grouping neighboring CpGs based on the genomic loci, we made two combinations, the HOXA9 promoter CpG (H9) group and the HOXA10 promoter CpG (H10) group. After calculating the risk scores (RSs) of H9 and H10 based on the Cox proportional hazards model, we performed survival analyses with the previously used TCGA paired sample dataset (*n* = 82). We divided the patients into two groups based on the median of the Δ*β* value. The RS of the separate H9 and H10 combinations significantly predicted poor survival, but it did not better predict poor survival compared with the single CpG methylations shown in Fig. 2b (Fig. 4a top and middle). However, the RS of all CpG (H9 + H10) combinations highly significantly predicted and better predicted poor survival compared with the single CpG methylations shown in Fig. 2b (*p* = 1.9 × 10^−4^, *n* = 82, log-rank test) (Fig. 4a bottom).

The TCGA breast cancer dataset contains information about more than paired samples, but they do not have paired normal samples. However, this dataset could be used to evaluate the robustness of the combination of H9 and H10. In the case of this dataset, it is impossible to calculate the Δ*β* value because of the lack of paired normal samples. Thus, we divided the patient into two groups based on the median of the *β* value (methylation ratio) of the tumor samples. After the missing data were removed, the Infinium HM450k data and survival data of 781 patients were available (TCGA tumor). Using the dataset, we evaluated the prognostic abilities of the combinations of H9 and H10. Performing the same analysis as for Fig. 4a, we found that the RS of all CpG (H9 + H10) combinations showed better prognostic ability than H9 or H10 alone (*p* = 7.1 × 10^−5^, *n* = 781, log-rank test) (Fig. 4b). Additionally, we performed survival analysis using another dataset from the NCBI GEO database (GSE39004). For survival analysis, we divided the patients into two groups based on the median of the *β* value of tumor samples. The RS of all CpG (H9 + H10) combination also significantly predicted poor survival in the GSE39004 dataset (*p* = 2.1 × 10^−3^, *n* = 62, log-rank test) (Fig. 4c). Thus, we hypothesize that the combination of HOXA9 and HOXA10 promoter CpG methylation markers are enhanced, robust prognostic markers.

### Subtype independency of the combination of HOXA9 and HOXA10 methylation markers

Breast cancer patients are divided into several molecular subtypes based on the expression level of hormone receptors, such as ER positive, PR positive, Her2 positive, and triple negative [2]. Using Infinium HM450k data and clinical information, including molecular subtype markers in the TCGA tumor dataset, we divided the patients into molecular subtype groups. Breast cancer patients can also be divided into several molecular subtypes based on a well-known gene expression signature (PAM50) [26]. We also divided the patients of the TCGA tumor dataset into PAM50 subtype groups. Then, we examined the association between subtypes and the combination of HOXA9 and HOXA10 promoter CpG methylations (RS). RS also separated the poor survival patients in each subtype except the Her2-positive type in both the TCGA tumor and GSE39004 datasets (Additional file 1: Figure S3). For the Her2-positive type, we observed a similar tendency as for the other subtypes, but this tendency was not significant because of the small number of patients (*n* = 46). To assess the value of the combination of the HOXA9 and HOXA10 promoter CpG methylation markers as a prognostic marker for breast cancer, we performed a multivariate Cox proportional hazards analysis with other subtype markers. For multivariate analysis, we selected patients from TCGA tumor dataset who had a distinct subtype annotation as positive or negative (*n* = 249). The RS better predicted poor survival in the TCGA tumor dataset than for any other marker (Table 1). For validation, we performed the same analysis using the GSE39004 dataset after removing samples with missing values (*n* = 58). The RS also better predicted poor survival in the GSE39004 than any other markers (Table 2). Thus, we suggest that the combination of HOXA9 and HOXA10 methylation markers is an independent prognostic marker for breast cancer.

**Table 1:** Multivariate Cox proportional hazards analysis for the prediction of breast cancer patient survival (TCGA tumor) (HR: hazard ratio, CI: confidence interval)

Variable	Survival	
	HR (95% CI)	*p* value
Risk score (high vs. low)	9.01 (1.59–50.9)	0.0128
HER2 (positive vs. negative)	8.41 (1.39–51.1)	0.0207
Ductal vs. lobular	11.7 (0.00867–0.842)	0.0351
Stage (I, II vs. III, IV)	3.86 (0.815–18.3)	0.0888
PR (positive vs. negative)	15.0 (0.656–344)	0.0898
Age (over vs. under 55)	3.01 (0.793–11.4)	0.105
ER (positive vs. negative)	0.277 (0.0105–7.32)	0.443
PAM50		
Luminal A	Reference	–
Basal	8.23 (0.383–177)	0.178
HER2	6.72 (0.353–128)	0.205
Luminal B	0.726 (0.116–4.54)	0.732

*HR* hazard ratio, *CI* confidence interval

**Table 2:** Multivariate Cox proportional hazards analysis for the prediction of breast cancer patient survival (GSE39004)

Variable	Survival	
	HR (95% CI)	*p* value
Risk score (high vs. low)	3.23 (1.37–7.60)	0.00721
Age (over vs. under 55)	2.29 (0.938–5.57)	0.0688
ER (positive vs. negative)	0.413 (0.158–1.08)	0.0703
Stage (I, II vs. III, IV)	2.03 (0.840–4.93)	0.115
Triple negative or basal-like (yes vs. no)	1.05 (0.341–3.26)	0.927

*HR* hazard ratio, *CI* confidence interval

## Discussion

Many CpG methylations have been identified as diagnostic or prognostic markers for cancer [13, 14]. Some of them have been used in the clinical field [11, 12]. The methylation of CpGs on promoters has been known to inhibit the gene expression of the nearest target. Recently, the function of trans or long-range actions of CpG methylation has been revealed by genome-wide scale analyses [19], but distal CpGs are still excluded from methylation marker development because of the difficulty in defining CpG-target gene relationships. The trans or long-range action of the CpG-target gene interaction can be specified by chromatin interactome data, and Pol II ChIA-PET data can specify transcriptional regulation. To define the relationship between CpG and long-range target genes, we used Pol II ChIA-PET data. After testing the diagnostic and prognostic maker value, we selected several long-range CpG-gene interactions that could play an important role in breast cancer tumorigenesis and progression. The representative markers are the CpGs on the HOXA9 and HOXA10 promoters.

Homeobox (HOX) genes are highly conserved gene clusters that encode transcription factors mediating the development process [21–23]. In humans, HOX genes are divided into four clusters, HOXA through HOXD [27]. The HOX gene loci are reported to form three-dimensional nuclear structures through long-range chromatin interactions [28–31]. An association has been reported between the methylation of several CpGs on HOXA loci and breast cancer progression [32, 33]. The expression of many HOX genes is associated with tumorigenesis and cancer progression in various cancers [34–37]. In breast cancer, HOXA9 and HOXA10 act as tumor suppressor genes [21–23]. The methylation of the HOXA9 and HOXA10 promoters is associated with cancer progression in various cancers [24, 25]. These associations imply that studies of HOXA loci will provide good models for the long-range interactions between distal CpG methylation markers and target genes. According to our analysis of the correlation between CpG methylation and target gene expression, HOXA10 promoter CpGs showed a different correlation pattern with HOXA9 promoter CpGs. HOXA9 promoter CpG methylation tended to correlate with HOXA9 gene expression, but HOXA10 promoter CpG methylation tended to correlate with long-range HOXA9 gene expression rather than the expression of the nearer HOXA10 gene. In other words, the HOXA10 promoter had an enhancer-like function, whereas the HOXA9 promoter had a promoter function. We suggest that the promoter-promoter interaction between HOXA9 and HOXA10 is important in breast cancer progression through the enhancer-like action of HOXA10 promoter CpGs.

To select both diagnostic and prognostic markers, we started marker selection from paired samples. In the case of the expression level of HOXA9 and HOXA10, there was the possibility of only a diagnostic marker but not a prognostic marker. This result could be caused by the instability of RNA markers. The HOXA9 and HOXA10 promoter methylation markers showed potential as both diagnostic and prognostic markers. The initial selection was performed based on the Δ*β* value, and the combination of the six HOXA9 and HOXA10 promoter methylation markers showed a highly significant prognostic value. Based on the tumor *β* value, the combination also showed a highly significant prognostic value in two independent datasets. Molecular markers that are clinically used can be applied to specific subtypes, such as node-negative and ER-positive breast cancer [4, 5]. The combination of the HOXA9 and HOXA10 promoter methylation markers showed a subtype-independent effect. Multivariate analysis indicated that the combination acted like an independent variable in the prediction of the prognosis of breast cancer. Thus, we suggest that the long-range interplay of HOXA9 and HOXA10 promoter CpGs is an efficient and robust methylation marker for breast cancer.

Molecular markers using single gene expression or CpG methylation have been identified [11], but more marker panels of multiple genes or CpGs have been developed due to advantages in efficiency and robustness [4, 5]. Many multiple marker panels have been identified by unsupervised methods without considering biological mechanisms [12–14]. The HOXA9 and HOXA10 promoter CpGs were identified by a supervised method based on long-range chromatin interactions, and the detailed action is specified in the HOXA9 and HOXA10 transcriptional regulation module. With respect to translational and biological relevance, this method has advantages for specifying therapeutic targets and strategies. We suggest that our method provides a comprehensive and novel approach for the development of molecular markers for personalized medicine and facilitating the precise determination of cancer prognosis.

## Conclusions

Breast cancer is both the most common cancer and the most frequent cause of cancer-related deaths among women. Mammography and some molecular markers have been used for its diagnosis and prognosis, but these techniques have limitations in premenopausal breast cancer or specific subtypes. In this study, we show that a combination of HOXA9 and HOXA10 promoter methylation markers is significantly associated with the prognosis of breast cancer patients in independent datasets and compose a transcriptional regulation module through long-range chromatin interactions. In contrast to other clinically used methylation markers applied to specific subtypes, the combination of the HOXA9 and HOXA10 promoter methylation markers showed a subtype-independent manner. Therefore, we suggest that the prognostic model using the HOXA9 and HOXA10 promoter CpG combination has translational potential to facilitate determination of breast cancer prognosis and therapeutic strategies targeting a specific molecular regulation module.

## Additional file

Additional file 1: Figure S1. Estimation of HOXA9 and HOXA10 expression as diagnostic and prognostic markers for breast cancer. **Figure S2.** Validation of chromatin interaction between HOXA9 and HOXA10 promoters using 3C PCR in MDA-MB-231 breast cancer cells and MCF10A normal breast cells. **Figure S3.** Association of HOXA9 and HOXA10 methylation marker combinations and survival in various patient subtype groups. **Table S1.** Primers used in the study. (DOCX 218 kb)

## Abbreviations

3C: Chromosome conformation capture
5-aza C: 5-azacytidine
affyU133P2: Affymetrix Human Genome U133 Plus 2.0 microarray
ATCC: American Type Culture Collection
ChIA-PET: Chromatin interaction analysis by paired-end tag sequencing
CpG: A region of DNA where a cytosine nucleotide is followed by a guanine nucleotide
DMEM: Dulbecco’s modified Eagle medium
DMSO: Dimethyl sulfoxide
ENCODE: Encyclopedia of DNA Elements
ER: Estrogen receptor
GAPDH: Glyceraldehyde 3-phosphate dehydrogenase gene
GEO: Gene Expression Omnibus
Her2: Human epidermal growth factor receptor 2
HOXA10: Homeobox gene A 10
HOXA11: Homeobox gene A 11
HOXA9: Homeobox gene A 9
ICGC: International Cancer Genome Consortium
Infinium HM450k: Illumina Infinium Human Methylation 450 k BeadChip microarray
NCBI: National Center for Biotechnology Information
Pol II: RNase polymerase 2
PR: Progesterone receptor
RMA: Robust Multi-array Average
RNA-seq: High-throughput RNA sequencing
RPKM: Reads per kilobase per million mapped reads
RS: Risk score
TCGA: The Cancer Genome Atlas
TSS: Transcription start site
