# Supplementary material for: A long-range interactive DNA methylation marker panel for the promoters of HOXA9 and HOXA10 predicts survival in breast cancer patients
Source: Clin Epigenetics. 2017 Jul 24;9:73. doi: 10.1186/s13148-017-0373-z (PMC5525292; doi:10.1186/s13148-017-0373-z)
Supplement: Additional file 1: Figure S1. — Estimation of HOXA9 and HOXA10 expression as diagnostic and prognostic markers for breast cancer. Figure S2. Validation of chromatin interaction between HOXA9 and HOXA10 promoters using 3C PCR in MDA-MB-231 breast cancer cells and MCF10A normal breast cells. Figure S3. Association of HOXA9 and HOXA10 methylation marker combinations and survival in various patient subtype groups. Table S1. Primers used in the study. (DOCX 218 kb) [file 13148_2017_373_MOESM1_ESM.docx]

**Supplementary Figure**


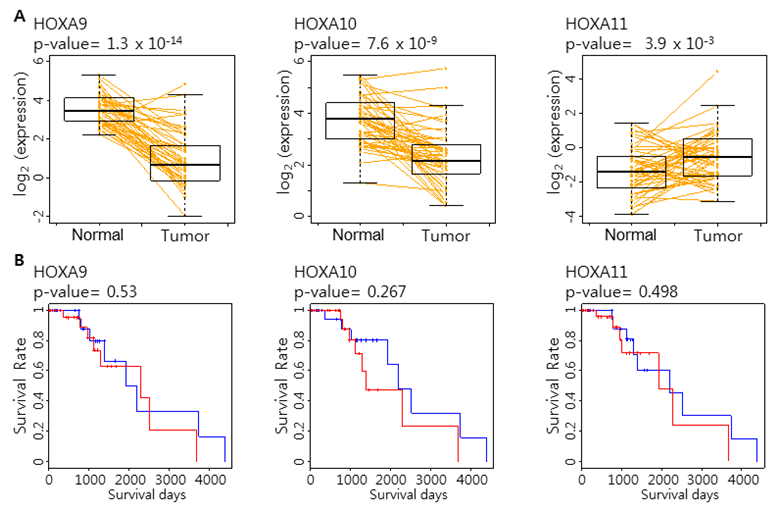


Supplementary Figure 1. Estimation of HOXA9 and HOXA10 expression as diagnostic and prognostic markers for breast cancer. A. Differences in expression between tumor and paired normal tissues. B. Association between survival and expression levels (red: high, blue: low).


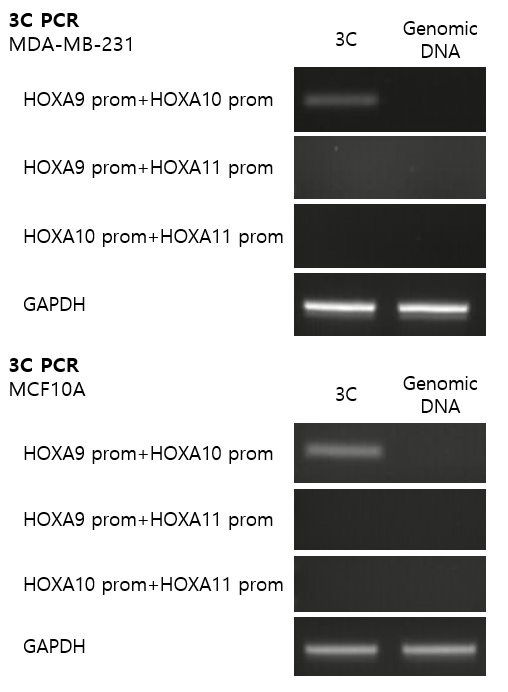


Supplementary Figure 2. Validation of chromatin interaction between HOXA9 and HOXA10 promoters using 3C PCR in MDA-MB-231 breast cancer cells and MCF10A normal breast cells


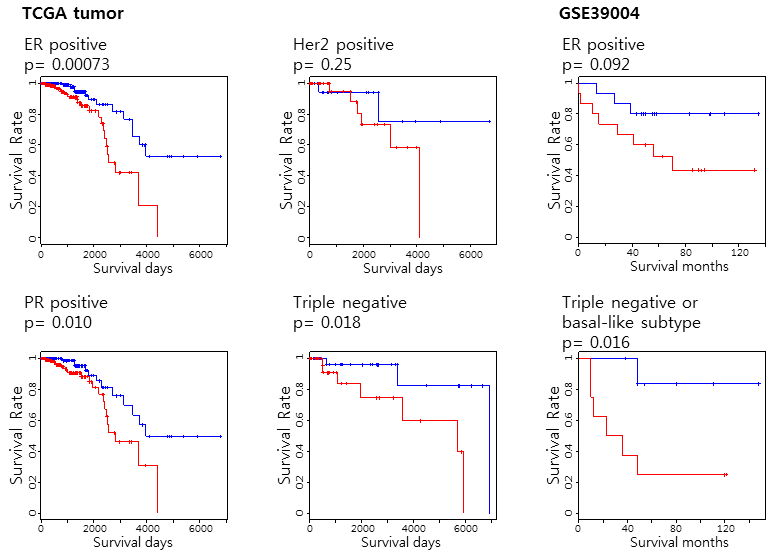


Supplementary Figure 3. Association of HOXA9 and HOXA10 methylation marker combinations and survival in various patient subtype groups (red: high, blue: low).

**Supplementary Table**

Supplementary Table 1. Primers used in the study (F: forward, R: reverse, S: sequencing)

| **Name** | **Sequence** |
| --- | --- |
| **3C PCR** |  |
| 3C_HOXA9_prom | CCCACTCCTTATTTAACCTTCC |
| 3C_HOXA10_prom | CCACTCCCACTCCCAAGG |
| 3C_HOXA11_prom | ACTTCCGAAGCGCTTTAGTG |
| GAPDH (internal) F | ACAGTCCATGCCATCACTGCC |
| GAPDH (internal) R | GCCTGCTTCACCACCTTCTTG |
| **RT-PCR** |  |
| GAPDH_F | TCCTGCACCACCAACTGCTTAG |
| GAPDH_R | TGGTCATGAGTCCTTCCACGATAC |
| HOXA9_F | GCGCCTTCTCTGAAAACAAT |
| HOXA9_R | CAGTTCCAGGGTCTGGTGTT |
| HOXA10_F | CTCGCCGGAGAAGGATTC |
| HOXA10_R | TCACTTGTCTGTCCGTGAGG |
| HOXA11_F | GGCAGCAGAGGAGAAAGAGC |
| HOXA11_R | CAGCCGCTGGAGTCTTAGAG |
| **Pyrosequencing** |  |
| HOXA9_promoter_1_F | GTTAGTTTGTGTGGTTTTTGAAATAATAA |
| HOXA9_promoter_1_R | biotin- CCCCCATACACACACTTCTTAAA |
| HOXA9_promoter_1_cg03464573_S | ATTTTATGTGAGGGGT |
| HOXA9_promoter_2_F | TTGGGTAAGGTGATGGTTATTATTGG |
| HOXA9_promoter_2_R | biotin- AAAACTAACCCAAAATCCCC |
| HOXA9_promoter_2_cg26521404_S | GGGGTTTTGGGTAATT |
| HOXA10_promoter_1_F | TAGGGGAGGGGAGAGTTTAATGGT |
| HOXA10_promoter_1_R | biotin- ACCCTTTCTAACTAACATTTCTTATAC |
| HOXA10_promoter_1_cg16857858_S | ATATTAATAGAGGGTTTTGAGGG |
| HOXA10_promoter_1_cg16967880_S | GTTTAAGAAATTAAATTGGGAGT |
